# Crocetin as New Cross-Linker for Bioactive Sericin Nanoparticles

**DOI:** 10.3390/pharmaceutics13050680

**Published:** 2021-05-09

**Authors:** Sara Perteghella, Giovanna Rassu, Elisabetta Gavini, Antonella Obinu, Elia Bari, Delia Mandracchia, Maria Cristina Bonferoni, Paolo Giunchedi, Maria Luisa Torre

**Affiliations:** 1 Department of Drug Sciences, University of Pavia, Viale Taramelli 12, I-27100 Pavia, Italy; sara.perteghella@unipv.it (S.P.); elia.bari@unipv.it (E.B.); mariacristina.bonferoni@unipv.it (M.C.B.); marina.torre@unipv.it (M.L.T.); 2 PharmaExceed S.r.l., 27100 Pavia, Italy; 3 Department of Chemistry and Pharmacy, University of Sassari, Via Muroni 23/a, I-07100 Sassari, Italy; eligav@uniss.it (E.G.); aobinu@uniss.it (A.O.); pgiunc@uniss.it (P.G.); 4 Department of Molecular and Translational Medicine, University of Brescia, Viale Europa 11, I-25123 Brescia, Italy; delia.mandracchia@unibs.it

**Keywords:** silk sericin nanoparticles, crocetin, nose-to-brain, antioxidant, neurologic disorders

## Abstract

The nose-to-brain delivery route is used to bypass the blood–brain barrier and deliver drugs directly into the brain. Over the years, significant signs of progress have been made in developing nano-drug delivery systems to address the very low drug transfer levels seen with conventional formulations (e.g., nasal solutions). In this paper, sericin nanoparticles were prepared using crocetin as a new bioactive natural cross-linker (NPc) and compared to sericin nanoparticles prepared with glutaraldehyde (NPg). The mean diameter of NPc and NPg was about 248 and 225 nm, respectively, and suitable for nose-to-brain delivery. The morphological investigation revealed that NPc are spherical-like particles with a smooth surface, whereas NPg seem small and rough. NPc remained stable at 4 °C for 28 days, and when freeze-dried with 0.1% *w/v* of trehalose, the aggregation was prevented. The use of crocetin as a natural cross-linker significantly improved the in vitro ROS-scavenging ability of NPc with respect to NPg. Both formulations were cytocompatible at all the concentrations tested on human fibroblasts and Caco-2 cells and protected them against oxidative stress damage. In detail, for NPc, the concentration of 400 µg/mL resulted in the most promising to maintain the cell metabolic activity of fibroblasts higher than 90%. Overall, the results reported in this paper support the employment of NPc as a nose-to-brain drug delivery system, as the brain targeting of antioxidants is a potential tool for the therapy of neurological diseases.

## 1. Introduction

Neurologic disorders are the second leading cause of death globally and the most significant cause of disability-adjusted life years [1]. Unfortunately, in treating many neurologic disorders, such as age-related neurodegenerative diseases, Alzheimer’s disease, Parkinson’s disease, or multiple sclerosis, the effective delivery of drugs to the brain is often hampered by the presence of the blood–brain barrier (BBB). Indeed, the BBB separates the central nervous system from the systemic circulation and comprises endothelial cells without fenestration, endothelial tight junctions, efflux pumps on the endothelial cells, and endothelial cells with low transcytotic capacity that overall limit the passage of most compounds from the blood to the brain [2].

Since the 1990s [3], the nose-to-brain delivery route has been proposed to bypass the BBB, exploiting the absorption and subsequent delivery of drugs directly into the brain via the olfactory neurons. In detail, for drug delivery, the olfactory region in the nasal cavity is exploited. The olfactory region has a surface area of about 2.5–10 cm^2^ in humans and a well-vascularized nasal epithelium, where the olfactory neurons are exposed [4]. Despite the exact mechanism by which compounds transfer from the nasal mucosa to the brain have to be fully understood yet, the nose-to-brain drug delivery seems to involve an intracellular or extracellular pathway. In the intracellular pathway, the compound is endocytosed by olfactory cells in the olfactory region, transported through the axon to the olfactory bulb’s synapses, and finally exocytosed. Conversely, in the extracellular pathway, the drug directly passes through paracellular spaces across the nasal epithelium directly into the cerebral spinal fluid [5]. In both cases, the drug has to reach the olfactory region of the nasal cavity in an appropriate concentration, being retained for a sufficient amount of time, dissolve, and being absorbed. Over the years, significant signs of progress have been made in terms of formulation to address the very low drug transfer levels seen with conventional formulations (e.g., nasal solutions). Specifically, nano-drug delivery systems have been developed in various forms (e.g., polymeric nanoparticles, solid lipid nanoparticles, nanoemulsions, and transfersomes) to enhance the penetration and increase the residence time of the drug in the nasal cavity [6,7,8,9,10].

In this work, silk sericin nanoparticles were developed as drug delivery systems for nose-to-brain delivery. Silk sericin is a water-soluble and globular protein derived from *Bombyx mori* cocoons. Until a few years ago, silk sericin was considered a waste material of the textile industry [11]. Some of its important biological properties have only recently been identified and, therefore, it is now considered a biomaterial suitable for potential wide use in pharmaceutical and cosmetic fields. The most important and well-known sericin activities are ROS-scavenging, anti-tyrosinase, anti-elastase, and in vitro immunomodulatory activities [12,13], as well as antibacterial, anti-inflammatory, and regenerative effects [14,15]. Therefore, due to these many intrinsic properties, sericin can be considered an excellent ingredient and a bioactive carrier of drugs, particularly for nanoparticle formulations [16,17]. Nevertheless, one of the limits to the use of sericin for nanoparticle formulation is related to the protein’s physical–chemical characteristics; in fact, sericin alone forms unstable nanoparticles because, in an aqueous solution, the nanostructures disassemble due to its hydrophilicity [15]. Therefore, the crosslinking agents or the blending with other stabilizer polymers is required [18,19,20,21,22,23,24]. Due to the amino acid composition, sericin can interact with different cross-linkers. Glutaraldehyde is the most used, but because of its toxicity, natural ones are preferable [15]. Genipin, from fruits of *Gardenia jasminoides Ellis*, has been proposed as a natural and non-toxic cross-linker for obtaining sericin nanoparticles [21] as well as chitosan [22].

In the present paper, crocetin is proposed as a new bioactive natural cross-linker for sericin nanoparticle formulation. Crocetin is a carotenoid present in the saffron (*Crocus sativus* L.) and exhibits various important therapeutic effects. It shows antitumor, neuroprotective, cardioprotective, hepatoprotective, antidepressant, antidiabetic, anti-inflammatory, anti-hyperlipidemia, and antiangiogenetic effects [25]. Moreover, it can inhibit β-amyloid (Aβ) fibril formation and improve Aβ degradation in vitro [26]. Crocetin is the product of glycosides hydrolysis of crocin, a diester of the disaccharide gentiobiose. In vivo, the hydrolysis occurs before or during intestinal absorption and, thus, crocetin results the in vivo bioactive compound [25]. However, crocetin is insoluble in water [27], limiting the oral bioavailability and therapeutic application. Recently, different nanoparticles were studied to improve crocetin’s solubility and efficacy [28,29,30,31,32].

Therefore, in this work, sericin/crocetin nanoparticles were proposed as nose-to-brain drug delivery systems. After nasal administration, crocetin could reach the brain directly and exert the neuroprotective effect enhanced by the antioxidant activity of sericin. A new formulation with crocetin (NPc) has been developed and compared to the standard glutaraldehyde formulation (NPg) in terms of physical-chemical properties, ROS scavenging activity, cytocompatibility, and cytoprotective effect against oxidative stress. Caco-2 cells and Human Fibroblasts were chosen as cell models. Caco-2 cells were selected as epithelial-type cells to mimic the structure of respiratory and olfactory epithelia of the nasal cavity [10,33]. Human fibroblasts were used as neuronal-type cells. In fact, fibroblasts were often chosen instead of brain cells due to the high difficulty in primary glial and neuronal cells culture [34]. Furthermore, fibroblasts usually are in the olfactory bulb, olfactory mucosa, and meninges [33]. Finally, a recent study shows that fibroblast could be a cell model of Alzheimer’s disease. Moreover, fibroblasts could reflect the metabolic changes produced in the brain Alzheimer’s disease [35].

## 2. Materials and Methods

### 2.1. Materials

The 2,2-diphenyl-2-picrylhydrazyl hydrate (DPPH), 3-(4,5-dimethylthiazol-2-yl)-2,5-diphenyltetrazolium bromide (MTT), ascorbic acid, crocin, D-(+)-dihydrate trehalose, dimethyl sulfoxide (DMSO), glutaraldehyde, and L-glutamine 200 mM were purchased from Sigma-Aldrich (Merck KGaA, Darmstadt, Germany). D-mannitol was bought from Roquette (Roquette Frères, Lestrem, France).

### 2.2. Silk Sericin Extraction

Silk sericin was extracted from *Bombyx mori* cocoons (poly hybrid strain), as previously reported [36]. Briefly, cocoons were cut and degummed in an autoclave (Systec V-65, Wurttemberg, Germany), at 120 °C for 1 h (40 mL water/g of cocoons). Sericin solution was then filtered by 70 μm cell strainers (Thermo Fisher Scientific, Milan, Italy) to eliminate the larger impurities, frozen at −80 °C and freeze-dried (Modulyo^®^ Edwards Freeze dryer, Kingston, New York, NY, USA) at 8 × 10^−1^ mbar and −50 °C for 72 h. The obtained sericin powder was stored at −20 °C until use. Sericin solution (5 mg/mL), used for all experiment, was prepared by solubilizing sericin powder in MilliQ water under magnetic stirring at 70–80 °C for 2 h. After that, the solution was centrifuged (Eppendorf Centrifuge 5702 R, Eppendorf, Hamburg, Germany) for 10 min at 4400 rpm and 4 °C to separate the insoluble impurities.

### 2.3. Preparation of Sericin Nanoparticles Using Glutaraldehyde as Cross-Linking Agent (NPg)

Sericin/glutamine nanoparticles (NPg) were prepared by the ethanol desolvation technique proposed by Das and co-workers [19], appropriately modified. Four mL of ethanol were dripped into 2 mL sericin solution (5 mg/mL), and then 1 mL of glutaraldehyde (2% *v/v*) was dripped to the suspension of the desolvated sericin. Glutamine (1 mL of 5 mg/mL solutions) was added to inactivate the free glutaraldehyde molecules. The nanoparticle suspension was centrifuged (Eppendorf Centrifuge 5702 R, Eppendorf, Hamburg, Germany) at 4400 rpm for 10 min and 4 °C. The supernatant was decanted and centrifuged until any pellet did not appear. The pellet was re-suspended in 1 mL MilliQ water by sonication with ultrasonic probe (Sonics & Materials, Inc., Danbury, CT, USA) for 10 min at 40% of amplitude.

### 2.4. Preparation of Sericin Nanoparticles Using Crocetin as Cross-Linking Agent (NPc)

**Figure 1:**
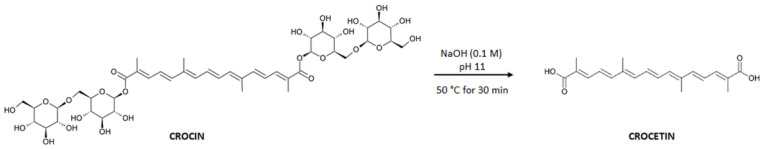
Chemical conversion of crocin to crocetin.

Sericin/crocetin nanoparticles (NPc) were prepared by adding 48 mg of crocin to 2 mL of sericin solution (5 mg/mL) under magnetic stirring; then, 4 mL of ethanol were dripped. The pH of the suspension was adjusted with NaOH (0.1 M) to reach pH 11. Then, the flask was covered with aluminum foil and heated 50 °C for 30 min to promote the hydrolysis of the sugar groups of crocin and liberate the crocetin (Figure 1). Afterwards, 1 mL of glutamine (5 mg/mL) was added drop by drop, and the suspension was centrifuged at 4400 rpm and 4 °C for 10 min. The supernatant was decanted to another tube and centrifuged again. Then, the pellet was solubilized with 1 mL of MilliQ water and sonicated with an ultrasonic probe at 40% of amplitude for 10 min.

This procedure was set up during the preformulation studies, where the influence of the addition method of the cross-linker and the amount of crocin (10, 20, and 48 mg) were evaluated. The cross-linker concentrations were chosen considering those of genipin proposed by Kanoujia et al. [21].

### 2.5. Determination of Nanoparticle Yield

After NPc and NPg preparation, the decanted supernatants were evaporated in the rotary evaporator (Büchi Rotavapor R110, Labortechnik AG, Flawil, Switzerland) at 80 °C under vacuum. The powder was then treated with 10 mL of HCl 0.2 M; the acidic solution solubilized sericin and crocetin, whereas crocin and glutamine were insoluble. The suspension was centrifuged at 4400 rpm and 4 °C for 15 min. The supernatant was evaporated, and then 1 mL of Milli-Q water was added; the flask was put in a water bath at 70 °C for 60 min to obtain the solubilization of sericin. Sericin solution was divided from crocetin by centrifugation at 4400 rpm and 4 °C for 15 min. The supernatant was dried and stored overnight in an oven (ULM4000, Memmert GmbH, Schwabach, Germany). The flask and the solid were weighted.

The yield percentage was calculated using the following equation analogously to [37]:

(1) $$Yield\;\left(\%\right)=\frac{\;sericin_{in}-sericin_{out}}{\;sericin_{in}}\times 100$$

where $sericin_{in}$ is the initial amount (mg) of sericin used for nanoparticles preparation and $sericin_{out}$ is the free sericin recovered in the supernatant.

### 2.6. Physical–Chemical Characterization of Sericin Nanoparticles

Both nanoparticle formulations have been characterized as follows.

#### 2.6.1. Particle Size, Size Distribution

The mean hydrodynamic diameter and the polydispersity index (PDI) of NPc and NPg in water were determined by photon correlation spectroscopy (PCS) using a Coulter N5 Submicron Particle Sizer Analyzer (Beckman-Coulter Inc. Particle Characterization, Miami, FL, USA). Three replicates of each formulation were analyzed.

#### 2.6.2. Morphological Characterization by Transmission Electron Microscope (TEM)

The morphology of nanoparticles was observed under Transmission Electron Microscope (TEM, Tecnai^®^ G2 F20 Twin TMP, FEI Company, Dawson Creek, Hillsboro, OR, USA). Samples were prepared by putting NPc and NPg (20 µL) on the carbon film cooper grid (200 mesh) and dried overnight at room temperature. Then, samples were stained with 1 M uranyl acetate solution and, after drying overnight, examined. TEM pictures were captured by using Digital Micrograph from Gatan and TIA software.

#### 2.6.3. Topography Characterization by Atomic Force Microscope (AFM)

Nanoparticle topography was analyzed using an atomic force microscope (AFM, MFP-3D Asylum Research, Oxford Instruments company, Santa Barbara, CA, USA) with tips from nanosensors model EFM (k$\frac{1}{4}$2N m_1, Ptlr5 coating). The samples were placed on a microscope glass slide and dried overnight before the analysis. Images were taken by Igor Pro6.3.4.1 MFP3D Template software.

#### 2.6.4. Fourier Transform Infrared (FTIR) Spectroscopy

The chemical structure of NPc was investigated by Fourier transform infrared (FTIR) spectroscopy (Avatar 320 FT-IT, Thermo Nicolet, Madison, WI, USA). Immediately after preparation, the pellet was washed six times with MilliQ water by centrifugation at 13,000 rpm for 10 min (Microcentrifuge Mikro 120, Hettich Zentrifugen, Tuttlingen, Germany) and then dried at room temperature. KBr pellet method was used to prepare samples. For comparison, FTIR spectra of NPc, crocetin and sericin were scanned by EzOminic version 6.0 software.

The crosslinking of sericin with crocetin was confirmed by thin-layer chromatography. Sericin and crocetin aqueous solution and crocetin and NPc solubilized in acetone were loaded on the TLC silica plates; the latter were placed in the chamber containing chloroform and methanol in the 9/1 *v/v* ratio and then air-dried and observed under a UV lamp. The Rf values were calculated.

### 2.7. Stability Studies

The stability of NPc and NPg as a function of time and temperature was evaluated by measuring the size and PDI of dispersions, stored at room temperature, or 4 °C, at 7, 14, and 28 days after preparation.

### 2.8. Lyophilization of NPc Nanoparticles

The NPc dispersion was freeze-dried with cryoprotective agents (Saez et al., 2000). D-mannitol (0.5% *w/w*) or D-(+)-dihydrate trehalose (0.5, 0.1 and 0.05% *w/w*) were added to NPc dispersion. Samples were frozen at −80 °C and freeze-dried (Lio5P series 030/2000, 5 Pascal, Milan, Italy). Samples were re-suspended in MilliQ water, and then the size and PDI of nanoparticles were measured, as reported in Section 2.6.

### 2.9. ROS-Scavenging Activity Assay

The ROS-scavenging activity of sericin-based nanoparticles was evaluated by the DPPH (2,2-diphenyl-2-picrylhydrazyl hydrate) assay, according to [38,39]. Briefly, NPg and NPc were tested at three concentrations: 2.5, 5, and 10 mg/mL (aqueous suspensions). At each considered concentration, each sample was mixed with DPPH methanolic solution (0.028% *w/v*) in the ratio of 1:40; the obtained mixtures were incubated in the dark for 20 min and then centrifuged (3000× *g*, 10 min). Supernatants were spectrophotometrically analyzed at 517 nm using a microplate reader (Synergy HT, BioTek, Swindon, UK). Ascorbic acid was considered a positive control, while as a negative control, the reaction mixture composed of water and DPPH solution in the ratio of 1:40 was analyzed.

The percentage of ROS scavenging activity was calculated according to the following equation:

% activity = (A − B)/A × 100(2)

where A was the absorbance of the negative control and B was the absorbance of the sample. Analyses were performed in three replicates.

### 2.10. Cell Metabolic Activity Evaluation (MTT Assay)

Caco-2 cells were obtained from the European Collection of Authenticated Cell Cultures Cell Bank (ECACC, Salisbury, UK). Cells were in vitro cultured in Dulbecco’s Modified Eagle’s Medium (DMEM) High Glucose supplemented with 10% *v/v* fetal bovine serum, non-essential amino acids (NEAA) 100×, penicillin 100 U/mL, streptomycin 100 μg/mL, amphotericin 0.25 μg/mL, glutamine 4 mM, sodium pyruvate 1 mM.

For MTT assay, cells were seeded into a 96-well plate (4500 cells/cm^2^) and treated for 24 h (37 °C, 5% CO_2_) with sericin-based nanoparticles at four concentrations: 100, 200, 400, and 800 µg/mL. Cells were then washed with PBS and 100 μL of 3-(4,5-dimethylthiazol-2-yl)-2,5-diphenyltetrazolium bromide (MTT) solution (0.5 mg/mL) were added to each well. After three incubation hours, the MTT solution was removed, and 100 μL of DMSO was added. Untreated cells were considered as control. Optical density was measured on Synergy HT at 570 nm and 670 nm (reference wavelength). Each condition was tested in triplicate, and the cell metabolic activity % was calculated as:

Cell metabolic activity % = 100 × (Abs_sample_/Abs_control_) (3)

where Abs_sample_ is the mean value of the measured absorbance of the tested samples, and Abs_control_ is the mean value of the measured absorbance of cells not incubated with nanoparticles.

### 2.11. Cytoprotective Effect against Oxidative Stress

Human Fibroblasts and Caco-2 cells were seeded in a 96-well plate (5000 cells/cm^2^) and treated with sericin-based nanoparticles (100, 200, 400, and 800 µg/mL). After 24 h of incubation, cells were washed with PBS and treated with hydrogen peroxide (H_2_O_2_, 1 mM) for 24 h. An MTT assay was then performed to evaluate the cell metabolic activity as reported in Section 2.10. Untreated cells were considered as control. Each condition was tested in triplicate.

### 2.12. Statistical Analysis

Data of stability studies were analyzed using one-way ANOVA followed by Dunnett’s test (GraphPad Prism, version 9.0.2; GraphPad Software Incorporated, San Diego, CA, USA). Other raw data were analyzed using STATGRAPHICS XVII (Statpoint Technologies, Inc., Warrenton, VA, USA). The raw data were processed using a linear generalized analysis of variance model (ANOVA) followed by Fisher’s least significant difference (LSD) procedure to estimate the differences between means. In detail, for the DPPH assay, the ROS-scavenging activity percentage was considered as the response variable and the sericin nanoparticle formulation and concentration as fixed factors. Cytotoxicity and oxidative stress data were processed considering the cell metabolic activity results as the response variable and the nanoparticle formulation and concentration as fixed factors; furthermore, for the oxidative stress protection results, the presence/absence of H_2_O_2_ was also considered as a fixed factor. Statistical significance was set at *p* < 0.05.

## 3. Results and Discussion

Sericin was selected as a carrier for the preparation of nanoparticles intended for the nose-to-brain delivery of drugs due to its intrinsic biological properties and excellent biocompatibility, non-immunogenicity and controllable biodegradability [40]. Unfortunately, sericin is physicochemically unstable; thus, nanoparticles’ preparation requires crosslinking, blending, or combining other polymers/compounds [41]. In this regard, this work demonstrated that it is possible to prepare sericin nanoparticle using crocetin as a crosslinking agent (NPc) instead of the standard glutaraldehyde (NPg). In detail, the preliminary studies highlighted that it is necessary to use a cross-linker for obtaining homogeneous nanoparticles in size (Supplementary Table S1). Furthermore, it was revealed that crocin’s hydrolysis into crocetin must occur in situ to form crosslinked sericin nanoparticles. In fact, a few and heterogeneous nanoparticles were obtained when free crocetin has been added to the sericin solution. Moreover, the highest amount of crocin (48 mg) resulted in optimal getting a homogeneous system. Glutamine is important to inactivate any free cross-linker molecules and ensure good stability to nanoparticle suspensions. The applied conditions reported for the preparation of NPc permitted to obtain reproducible nanoparticle formulations.

**Figure 2:**
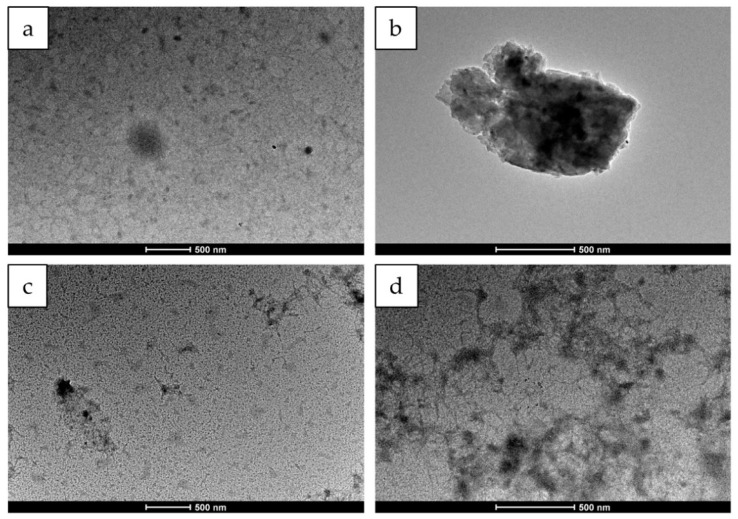

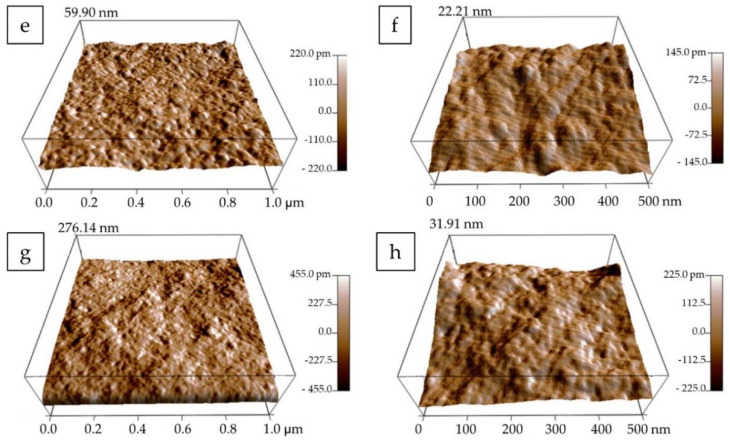
TEM pictures of NPc (**a**,**b**) and NPg (**c**,**d**). Scale bar: 500 nm. AFM images of NPc (**e**,**f**) and NPg (**g**,**h**).

The yield was calculated by determining the sericin’s residual amount in the supernatants after NPc and NPg preparation. The results show that the yield of NPc is 98.61 ± 1.03% versus 90.69 ± 2.06% of NPg. The physicochemical properties of NPc and NPg were then compared. After their production, the mean hydrodynamic diameter and polydispersity index were determined. The mean diameter is 248.33 ± 6.10 nm and 225.45 ± 10.70 nm of NPc and NPg, respectively (*p* > 0.05). The PI of NPc is 0.23 ± 0.05 and 0.23 ± 0.03 of NPg (*p* > 0.05). For both formulations, the size is appropriate for a nose-to-brain delivery. Indeed, following the earlier studies where it seemed that 100 nm is the largest nanoparticle size for nose-to-brain translocation [42,43], it was then demonstrated that nanoparticles could reach the brain upon intranasal administration even when up to 300–600 nm [44,45]. NPc appear morphologically well-defined with respect to NPg with an almost spherical shape Figure 2b. Filamentous structures are observed in NPg Figure 2d due to nanoparticle aggregation [19]. AFM images Figure 2e–f confirm that NPc are spherical-like particles with a smooth surface, whereas NPg seem small and rough Figure 2g–h. Overall, the particle size, morphology, and yield in the preparation of protein nanoparticles by a crosslinking method depend on the concentration and pH of the protein solution, the temperature, the desolvation agent, and the type of cross-linker [37]. In detail, glutaraldehyde crosslinks proteins by a nucleophilic attack: the amino groups of lysine and arginine residues in the protein react with two carbonyl groups of cross-linker, forming Schiff bases in solution [46]. Conversely, for crocetin, the crosslinking mechanism with protein has not been thoroughly investigated yet. Only for polysaccharides, Ali and colleagues demonstrated that crocin acts, by forming, in an aqueous solution, non-covalent interactions with different molecular structures [47]. Moreover, the higher yield obtained using crocetin as crosslinking agent likely indicates a more efficient crosslinking process, probably related to the basic pH used during the preparation process. Indeed, the amino groups of the protein are deprotonated in high pH, so further free amino groups are available for interaction with another group [37].

**Figure 3:**
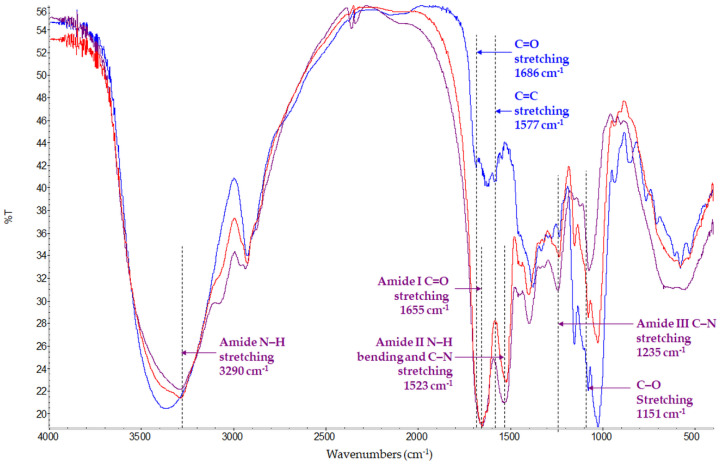
FT-IR spectra of NPc (red line), crocetin (blue line), and sericin (purple line).

Figure 3 shows the FTIR spectra of NPc, crocetin and sericin. NPc present the distinguishable peaks of sericin at 1655 cm^−1^ (amide I), at 1523 cm^−1^ (amide II), and at 1235 cm^−1^ (amide III) as well as at 3290 cm^−1^ (stretching of the N–H and O-H bonds); these peaks indicate that sericin in nanoparticles has β-sheet structure [23]. Crocetin shows the characteristic peaks at 1686 cm^−1^ and 1577 cm^−1^ attributable to the C=O stretching of two carboxylic groups and the C=C stretching in the carbon chain, respectively [26]. In NPc spectra, these peaks are covered by those of the sericin.

Thin-layer chromatography was employed to confirm the successful crosslinking of sericin with crocetin. Figure S1 shows the spots resulting from TLC analysis. Both sericin and crocin cannot migrate from the origin in the solvent system used, and spots were detected on the origin (Rf = 0). On the contrary, for both crocetin and NPc, yellow spots were recovered with Rf of 0.54 and 0.91, respectively. These results suggest that a new chemical entity originated from the reaction between sericin and crocetin, and it is undoubtedly different from the starting components. Therefore, it can be stated that the crocetin acts as a cross-linker for sericin nanoparticles formation.

**Figure 4:**
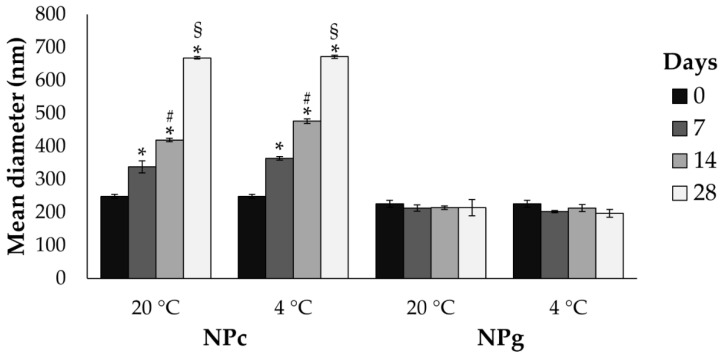
Particle size of NPc and NPg measured immediately after preparation (t0) and then after 7, 14, and 28 days, both stored at 20 °C and 4 °C. * *p* < 0.05 versus NPc t0; ^#^ *p* < 0.05 versus NPc t7 both at 20 °C and 4 °C; ^§^ *p* < 0.05 versus NPc t14 both at 20 °C and 4 °C; ^$^ *p* < 0.05 versus NPg t0.

After preparation, NPc and NPg were stored at room temperature and 4 °C and particle size and PDI were measured after 7, 14, 28 days to evaluate their physical stability. Figure 4 reports the variation of particle size as a function of time and temperature. The size of NPc increases gradually and significantly already during the first 7 days and up to 28 days of storage regardless of temperature. The PDI instead increase significantly after 14 days, reaching the value of 0.45 ± 0.07 and 1.07 ± 0.09 after 28 days at 20 °C (*p* < 0.05); no significant differences were observed in the samples stored at 4 °C (*p* > 0.05). On the contrary, NPg have good physical stability during the time regardless of the temperature of storage; rather, the mean diameter is slightly decreased after 7 days of storage at 4 °C (*p* < 0.05). The PDI significantly increases after 14 days (0.35 ± 0.07), reaching the values of 0.42 ± 0.10 after 28 days.

**Figure 5:**
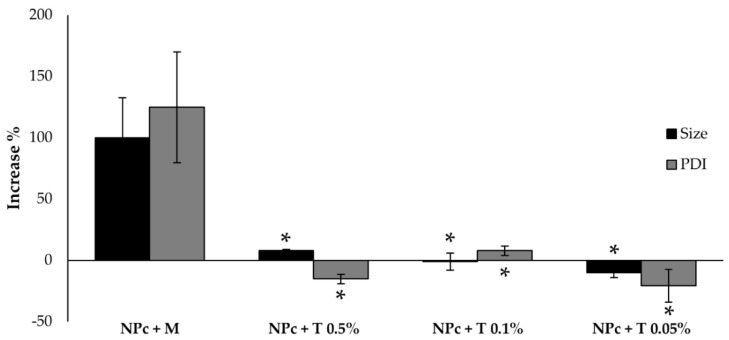
Changes in particle size and PDI of NPc after reconstitution of lyophilized powder containing 0.5% *w/w* mannitol (NPc + M), trehalose in concentration of 0.5% *w/w* (NPc + T 0.5%), 0.1% *w/w* (NPc + T 0.1%), and 0.05% *w/w* (NPc + T 0.05%). * *p* < 0.05 versus NPc + M.

Nanoparticles were freeze-dried to improve long-term physical stability. Cryoprotectants were used for avoiding severe aggregation during the drying process. Specifically, mannitol and trehalose were selected because they are the most commonly used cryoprotectants [48]. The results, reported in Figure 5, show that mannitol in the concentration of 0.5% *w/w* (NPc + M) cannot protect NPc from aggregation: in fact, both size and PDI of lyophilized nanoparticles after reconstitution increase significantly by 100%. On the contrary, trehalose effectively prevents aggregation even at low concentrations (*p* < 0.05).

**Figure 6:**
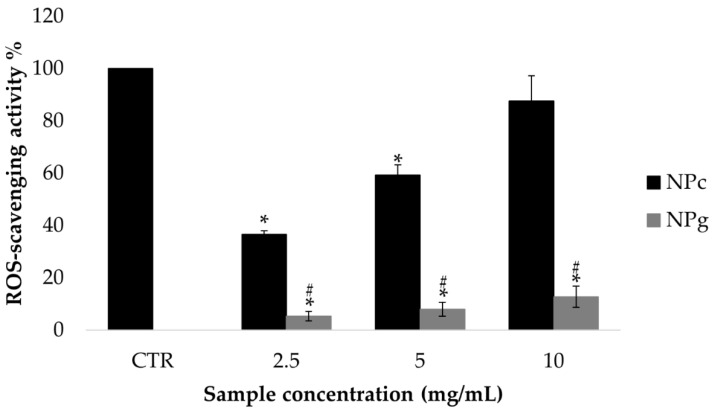
Reactive oxygen species (ROS) scavenging activity % as a function of nanoparticle concentrations (mg/mL). Results are reported as mean values ± standard deviation (*n* = 3). Ascorbic acid, considered a positive control, presented a ROS-scavenging ability of 100% at the concentration of 1.25 mg/mL. * *p* < 0.05 versus CTR; ^#^ *p* < 0.05 versus NPc.

The ROS-scavenging activity results are reported in Figure 6 for both NPc and NPg. The natural cross-linker use significantly improved (*p* < 0.05) the in vitro ROS-scavenging ability of NPc with respect to NPg. Indeed, NPc presented a dose-depended activity reaching a ROS-scavenging capacity of about 90% at the concentration of 10 mg/mL. On the other side, considering the NPg sample, no significant differences were observed testing the different concentrations, and the maximum antioxidant activity of 12% was reached at the highest tested dose. For NPg, the antioxidant properties are likely due to sericin, which is well known to possess ROS-scavenging activity. However, it seems that crosslinking with glutaraldehyde reduced sericin protein activity, as other papers reported a more substantial activity for sericin alone [23,49]. Conversely, NPc showed excellent ROS-scavenging properties, linked to the documented antioxidant effect of both crocetin [31] and sericin. Overall, such results are interesting for a nose-to-brain drug delivery system, as the brain targeting of antioxidants is a potential tool for the therapy of neurological diseases, as recently reviewed [50].

**Figure 7:**
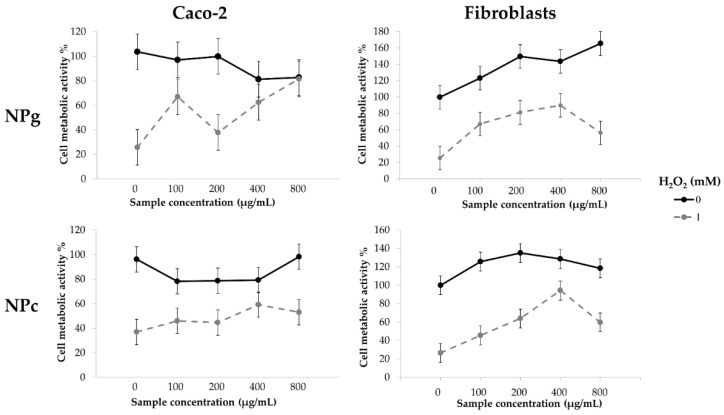
Cytoprotective properties of NPc and NPg on Caco-2 and human fibroblasts treated (grey dashed line) or not treated (continuous black line) with H_2_O_2_ 1 mM for 24 h. Multifactor ANOVA, mean values ± least significant difference (LSD), *n* = 3. The overlap of two LSD intervals graphically indicates the absence of significant differences (*p* > 0.05).

The cytocompatibility of NPg and NPc was evaluated on fibroblasts and Caco-2 cells considering four sample concentrations: 100, 200, 400, and 800 µg/mL. Both NPg and NPc resulted as cytocompatible; in fact, cells maintained a relative cell metabolic activity higher than 80% at all considered concentrations (Figure 7, black continuous line) with both samples. Statistical analysis performed on Caco-2 viability data demonstrated that both treatment (*p* = 0.0895) and sample concentration (*p* = 0.0566) had no significant effect on cell metabolic activity. Overall, Caco-2 cells treated with NPc and NPg presented a mean metabolic activity of 86.17 ± 12.55 and 92.93 ± 13.20%, respectively. On the other side, data obtained on human fibroblast cells demonstrated that treatment (*p* = 0.0047) and sample concentration (*p* < 0.0001) significantly affected the cell metabolic activity. Fibroblasts treated with NPg samples presented an increased cell metabolic activity for doses higher than 200 µg/mL (Figure 7, black continuous line). The same trend was observed for NPc samples that induce an increase of fibroblast metabolic activity at all considered concentrations (Figure 7, black continuous line). Increased cell metabolic activity following sericin treatment was previously reported and linked to the protein’s mitogen effect [23,36]. It is worth nothing that the use of crocin (instead of the cytotoxic glutaraldehyde) reflects the safety of the final product and reduces the exposure risk to harmful substances for the personnel operating in the manufacturing process.

Finally, the ability of NPg and NPc to protect human fibroblasts and Caco-2 cells from the damage induced by hydrogen peroxide was tested. The suitability of this in vitro model was demonstrated by the significant (*p* < 0.05) reduction of fibroblasts and Caco-2 viability after their treatment with H_2_O_2_ (1 mM) (Figure 7, grey dashed line, sample concentration 0 mg/mL). NPg were able to protect the Caco-2 cells by the oxidative stress damages; considering 100, 400, and 800 µg/mL sample concentrations, no significant differences (*p* < 0.05) were observed between treated and untreated cells (Figure 7, grey dashed and continuous black lines, respectively). The ability of NPc to protect the cells by the hydrogen peroxide effect was less than that observed with the NPg sample. In fact, the cell viability of damaged cells (Figure 7, grey dashed line) was significantly lower than the control cells (Figure 7, continuous black line). Furthermore, both nanoparticle formulations were able to protect human fibroblast from the damages induced by oxidative stress. Considering NPg formulation, all tested concentrations maintained the cell metabolic activity higher than 60%; in particular, no significant differences were evidenced between control cells (no nanoparticle treatment and no hydrogen peroxide treatment) and fibroblast incubated with NPg sample (200 and 400 µg/mL) (Figure 7). The use of a natural cross-linker for nanoparticle formulation allowed to obtain a nano-drug delivery system able to protect human fibroblast from the hydrogen peroxide-mediated damages. In particular, the concentration of 400 µg/mL resulted in the most promising to maintain the cell metabolic activity higher than 90% (Figure 7).

## 4. Conclusions

In this paper, sericin nanoparticles were prepared using crocetin as a new bioactive natural cross-linker (NPc) and compared to sericin nanoparticles prepared with glutaraldehyde (NPg). NPc and NPg had a diameter of about 248 and 225 nm, respectively. SEM and ATM analyses revealed that NPc are spherical-like particles with a smooth surface, whereas NPg seem small and rough. NPc remained stable at 4 °C for 28 days, and when freeze-dried with 0.1% *w/v* of trehalose, the aggregation was prevented. The use of crocetin as a natural cross-linker significantly improved the in vitro ROS-scavenging ability of NPc with respect to NPg, reaching an antioxidant activity of about 90% at a concentration of 10 mg/mL. Both formulations were cytocompatible at all the concentrations tested on human fibroblasts and Caco-2 cells and protected them against oxidative stress damage. In detail, for NPc, the concentration of 400 µg/mL resulted in the most promising to maintain the cell metabolic activity of fibroblasts higher than 90%. Overall, the results reported in this paper support the employment of NPc as a nose-to-brain drug delivery system, as the brain targeting of crocetin and antioxidants is a potential tool for the therapy of neurological diseases.

## Data Availability

The data presented in this study are contained within the article.

## Supplementary Materials

The following are available online at https://www.mdpi.com/article/10.3390/pharmaceutics13050680/s1. Table S1. Methods tested and result obtained during the preliminary studies, where the influence of the addition method of the cross-linker and the amount of crocin (10, 20, and 48 mg) were evaluated. Data are reported as mean ± standard deviation. Figure S1. TLC plate with spots related to sericin (A), crocin (B), crocetin (C), and NPc (D).

## References

[1] Feigin V.L., Vos T., Nichols E., Owolabi M.O., Carroll W.M., Dichgans M., Deuschl G., Parmar P., Brainin M., Murray C. (2020). The global burden of neurological disorders: Translating evidence into policy. Lancet Neurol..

[2] Ballabh P., Braun A., Nedergaard M. (2004). The blood-brain barrier: An overview—Structure, regulation, and clinical implications. Neurobiol. Dis..

[3] Frey W.H. (1991). Neurologic Agents for Nasal Administration to the Brain. U.S. Patent.

[4] Gizurarson S. (2012). Anatomical and histological factors affecting intranasal drug and vaccine delivery. Curr. Drug Deliv..

[5] Selvaraj K., Gowthamarajan K., Karri V. (2018). Nose to brain transport pathways an overview: Potential of nanostructured lipid carriers in nose to brain targeting. Artif. Cells Nanomed. Biotechnol..

[6] Wang Z., Xiong G.J., Tsang W.C., Schatzlein A.G., Uchegbu I.F. (2019). Nose-to-brain delivery. J. Pharmacol. Exp. Ther..

[7] Langasco R., Fancello S., Rassu G., Cossu M., Cavalli R., Galleri G., Giunchedi P., Migheli R., Gavini E. (2019). Increasing protective activity of genistein by loading into transfersomes: A new potential adjuvant in the oxidative stress-related neurodegenerative diseases?. Phytomedicine.

[8] Rassu G., Porcu E.P., Fancello S., Obinu A., Senes N., Galleri G., Migheli R., Gavini E., Giunchedi P. (2019). Intranasal delivery of genistein-loaded nanoparticles as a potential preventive system against neurodegenerative disorders. Pharmaceutics.

[9] Bonferoni M.C., Rossi S., Sandri G., Ferrari F., Gavini E., Rassu G., Giunchedi P. (2019). Nanoemulsions for “nose-to-brain drug“ delivery. Pharmaceutics.

[10] Rassu G., Soddu E., Posadino A.M., Pintus G., Sarmento B., Giunchedi P., Gavini E. (2017). Nose-to-brain delivery of BACE1 siRNA loaded in solid lipid nanoparticles for Alzheimer‘s therapy. Colloids Surf. B Biointerfaces.

[11] Orlandi G., Farago S., Menato S., Sorlini M., Butti F., Mocchi M., Donelli I., Catenacci L., Sorrenti M.L., Croce S. (2020). Eco-sustainable silk sericin from by-product of textile industry can be employed for cosmetic, dermatology and drug delivery. J. Chem. Technol. Biotechnol..

[12] Chlapanidas T.F.S., Lucconi G., Perteghella S., Galuzzi M., Mantelli M., Avanzini M.A., Tosca M.C., Marazzi M., Vigo D., Torre M.L. (2013). Sericins exhibit ROS-scavenging, anti-tyrosinase, anti-elastase, and in vitro immunomodulatory activities. Int. J. Biol. Macromol..

[13] Chlapanidas T., Perteghella S., Leoni F., Farago S., Marazzi M., Rossi D., Martino E., Gaggeri R., Collina S. (2014). TNF-alpha blocker effect of naringenin-loaded sericin microparticles that are potentially useful in the treatment of psoriasis. Int. J. Mol. Sci..

[14] Kunz R.I., Brancalhao R.M.C., Ribeiro L.D.F.C., Natali M.R.M. (2016). Silkworm sericin: Properties and biomedical applications. BioMed Res. Int..

[15] Das G., Shin H.S., Campos E.V.R., Fraceto L.F., Rodriguez-Torres M.D., Mariano K.C.F., de Araujo D.R., Fernandez-Luqueno F., Grillo R., Patra J.K. (2021). Sericin based nanoformulations: A comprehensive review on molecular mechanisms of interaction with organisms to biological applications. J. Nanobiotechnol..

[16] Aramwit P., Ekasit S., Yamdech R. (2015). The development of non-toxic ionic-crosslinked chitosan-based microspheres as carriers for the controlled release of silk sericin. Biomed. Microdevices.

[17] Khampieng T., Aramwit P., Supaphol P. (2015). Silk sericin loaded alginate nanoparticles: Preparation and anti-inflammatory efficacy. Int. J. Biol. Macromol..

[18] Mandal B.B., Kundu S.C. (2009). Self-assembled silk sericin/poloxamer nanoparticles as nanocarriers of hydrophobic and hydrophilic drugs for targeted delivery. Nanotechnology.

[19] Das S.K., Dey T., Kundu S.C. (2014). Fabrication of sericin nanoparticles for controlled gene delivery. RSC Adv..

[20] Parisi O.I., Fiorillo M., Scrivano L., Sinicropi M.S., Dolce V., Iacopetta D., Puoci F., Cappello A.R. (2015). Sericin/Poly(ethylcyanoacrylate) nanospheres by interfacial polymerization for enhanced bioefficacy of fenofibrate: In vitro and in vivo studies. Biomacromolecules.

[21] Kanoujia J., Singh M., Singh P., Saraf S.A. (2016). Novel genipin crosslinked atorvastatin loaded sericin nanoparticles for their enhanced antihyperlipidemic activity. Mater. Sci. Eng. C Mater. Biol. Appl..

[22] Hu D., Xu Z., Hu Z., Hu B., Yang M., Zhu L. (2017). pH-triggered charge-reversal silk sericin-based nanoparticles for enhanced cellular uptake and doxorubicin delivery. ACS Sustain. Chem. Eng..

[23] Orlandi G., Bari E., Catenacci L., Sorrenti M., Segale L., Farago S., Sorlini M., Arciola C.R., Torre M.L., Perteghella S. (2020). Polyphenols-loaded sericin self-assembling nanoparticles: A slow-release for regeneration by tissue-resident mesenchymal stem/stromal cells. Pharmaceutics.

[24] Giovannelli L., Milanesi A., Ugazio E., Fracchia L., Segale L. (2021). Effect of methyl-beta-cyclodextrin and trehalose on the freeze-drying and spray-drying of sericin for cosmetic purposes. Pharmaceuticals.

[25] Hashemi M., Hosseinzadeh H. (2019). A comprehensive review on biological activities and toxicology of crocetin. Food Chem. Toxicol..

[26] Wong K.H., Xie Y.N., Huang X., Kadota K., Yao X.S., Yu Y., Chen X.Y., Lu A.P., Yang Z.J. (2020). Delivering crocetin across the blood-brain barrier by using gamma-cyclodextrin to treat Alzheimer‘s disease. Sci. Rep..

[27] Bathaie S.Z., Farajzade A., Hoshyar R. (2014). A review of the chemistry and uses of crocins and crocetin, the carotenoid natural dyes in saffron, with particular emphasis on applications as colorants including their use as biological stains. Biotech. Histochem..

[28] Ghahestani Z.H., Langroodi F.A., Mokhtarzadeh A., Ramezani M., Hashemi M. (2017). Evaluation of anti-cancer activity of PLGA nanoparticles containing crocetin. Artif. Cells Nanomed. Biotechnol..

[29] Neyshaburinezhad N., Kalahnia F., Hashemi M. (2019). Encapsulation of crocetin into poly (lactic-co-glycolic acid) nanoparticles overcomes drug resistance in human ovarian cisplatin-resistant carcinoma cell line (A2780-RCIS). Mol. Biol. Rep..

[30] Pradhan J., Mohanty C., Sahoo S.K. (2019). Protective efficacy of crocetin and its nanoformulation against cyclosporine A-mediated toxicity in human embryonic kidney cells. Life Sci..

[31] Puglia C., Santonocito D., Musumeci T., Cardile V., Graziano A.C.E., Salerno L., Raciti G., Crasci L., Panico A.M., Puglisi G. (2019). Nanotechnological approach to increase the antioxidant and cytotoxic efficacy of crocin and crocetin. Planta Med..

[32] Yang X.D. (2019). Design and optimization of crocetin loaded PLGA nanoparticles against diabetic nephropathy via suppression of inflammatory biomarkers: A formulation approach to preclinical study. Drug Deliv..

[33] Erdo F., Bors L.A., Farkas D., Bajza A., Gizurarson S. (2018). Evaluation of intranasal delivery route of drug administration for brain targeting. Brain Res. Bull..

[34] Schuh R.S., Bidone J., Poletto E., Pinheiro C.V., Pasqualim G., de Carvalho T.G., Farinon M., Diel D.d.S., Xavier R.M., Baldo G. (2018). Nasal administration of cationic nanoemulsions as nucleic acids delivery systems aiming at mucopolysaccharidosis type I gene therapy. Pharm. Res..

[35] Perez M.J., Ponce D.P., Osorio-Fuentealba C., Behrens M.I., Quintanilla R.A. (2017). Mitochondrial bioenergetics is altered in fibroblasts from patients with sporadic Alzheimer‘s disease. Front. Neurosci..

[36] Bari E., Perteghella S., Marrubini G., Sorrenti M., Catenacci L., Tripodo G., Mastrogiacomo M., Mandracchia D., Trapani A., Farago S. (2018). In vitro efficacy of silk sericin microparticles and platelet lysate for intervertebral disk regeneration. Int. J. Biol. Macromol..

[37] Amighi F., Emam-Djomeh Z., Labbafi-Mazraeh-Shahi M. (2020). Effect of different cross-linking agents on the preparation of bovine serum albumin nanoparticles. J. Iran. Chem. Soc..

[38] Bari E., Arciola C.R., Vigani B., Crivelli B., Moro P., Marrubini G., Sorrenti M., Catenacci L., Bruni G., Chlapanidas T. (2017). In vitro effectiveness of microspheres based on silk sericin and chlorella vulgaris or arthrospira platensis for wound healing applications. Materials.

[39] Della Cuna F.S.R., Calevo J., Bari E., Giovannini A., Boselli C., Tava A. (2019). Characterization and antioxidant activity of essential oil of four sympatric orchid species. Molecules.

[40] Bari E., Perteghella S., Farago S., Torre M.L. (2018). Association of silk sericin and platelet lysate: Premises for the formulation of wound healing active medications. Int. J. Biol. Macromol..

[41] Crivelli B., Perteghella S., Bari E., Sorrenti M., Tripodo G., Chlapanidas T., Torre M.L. (2017). Silk nanoparticles: From inert supports to bioactive natural carriers for drug delivery. Soft Matter..

[42] Chiappetta D.A., Hocht C., Opezzo J.A.W., Sosnik A. (2013). Intranasal administration of antiretroviral-loaded micelles for anatomical targeting to the brain in HIV. Nanomedicine.

[43] Mistry A., Glud S.Z., Kjems J., Randel J., Howard K.A., Stolnik S., Illum L. (2009). Effect of physicochemical properties on intranasal nanoparticle transit into murine olfactory epithelium. J. Drug Target..

[44] Kanazawa T., Taki H., Tanaka K., Takashima Y., Okada H. (2011). Cell-penetrating peptide-modified block copolymer micelles promote direct brain delivery via intranasal administration. Pharm. Res..

[45] Mistry A., Stolnik S., Illum L. (2015). Nose-to-brain delivery: Investigation of the transport of nanoparticles with different surface characteristics and sizes in excised porcine olfactory epithelium. Mol. Pharm..

[46] Bronze-Uhle E.S., Costa B.C., Ximenes V.F., Lisboa P.N. (2017). Synthetic nanoparticles of bovine serum albumin with entrapped salicylic acid. Nanotechnol. Sci. Appl..

[47] Ali S., Davinelli S., Mencucci R., Fusi F., Scuderi G., Costagliola C., Scapagnini G. (2021). Crosslinked hyaluronic acid with liposomes and crocin confers cytoprotection in an experimental model of dry eye. Molecules.

[48] Mohammady M., Mohammadi Y., Yousefi G. (2020). Freeze-drying of pharmaceutical and nutraceutical nanoparticles: The effects of formulation and technique parameters on nanoparticles characteristics. J. Pharm. Sci..

[49] Tengattini S., Orlandi G., Perteghella S., Bari E., Amadio M., Calleri E., Massolini G., Torre M.L., Temporini C. (2020). Chromatographic profiling of silk sericin for biomedical and cosmetic use by complementary hydrophylic, reversed phase and size exclusion chromatographic methods. J. Pharm. Biomed. Anal..

[50] Bonferoni M.C., Rassu G., Gavini E., Sorrenti M., Catenacci L., Giunchedi P. (2020). Nose-to-brain delivery of antioxidants as a potential tool for the therapy of neurological diseases. Pharmaceutics.

