# Supplementary material for: Crocetin as New Cross-Linker for Bioactive Sericin Nanoparticles
Source: Pharmaceutics. 2021 May 9;13(5):680. doi: 10.3390/pharmaceutics13050680 (PMC8150760; doi:10.3390/pharmaceutics13050680)
Supplement: Supplementary file 1 [file pharmaceutics-13-00680-s001.zip › pharmaceutics-1210570-supplementary.pdf]

pharmaceutics

Supplementary Materials: Crocetin as New Cross-Linker for Bioactive Sericin Nanoparticles

Sara Perteghella, Giovanna Rassu, Elisabetta Gavini, Antonella Obinu, Elia Bari, Delia Mandracchia, Maria Cristina Bonferoni, Paolo Giunchedi and Maria Luisa Torre

Table 1. Methods tested and result obtained during the preliminary studies, where the influence of the addition method of the cross-linker and the amount of crocin (10, 20 and 48 mg) were evaluated. Data are reported as mean ± standard deviation.

Test n.	Sericin Solution (mL)	Crocin (mg)	Ethanol (mL)	Glutamine (mL)	Method	Particle Size and Size Distribution	
**1**	2	48	4 mL	1	1. Preparation of the sericin/crocin solution; 2. Desolvation with ethanol; 3. Heating at 50 °C for 30 min; 4. Addition of the glutamine solution.	Mean diameter (nm)	248.33 ± 6.10
						PI	0.23 ± 0.05
**2**	2	48	4 mL	1	1. Heating at 50 °C for 30 min of the crocin solution; 2. Preparation of the sericin solution and desolvation with ethanol; 3. Addition of the hydrolyzed crocin solution to the sericin solution; 4. Addition of the glutamine solution.	Mean diameter (nm)	344.73 ± 25.32
						PI	1.28 ± 0.05
**3**	2	48	4 mL	1	1. Preparation of the sericin/crocin solution; 2. Heating at 50 °C for 30 min; 3. Addition of the glutamine solution; 4. Desolvation with ethanol.	Mean diameter (nm)	294.20 ± 62.22
						PI	1.006 ± 0.67
**4**	2	20	4 mL	1	1. Preparation of the sericin/crocin solution; 2. Desolvation with ethanol; 3. Heating at 50 °C for 30 min; 4. Addition of the glutamine solution.	Mean diameter (nm)	404.07 ± 4.71
						PI	0.603 ± 0.12
**5**	2	10	4 mL	1	1. Preparation of the sericin/crocin solution; 2. Desolvation with ethanol; 3. Heating at 50 °C for 30 min; 4. Addition of the glutamine solution.	Mean diameter (nm)	517.43 ± 13.57
						PI	0.617 ± 0.08

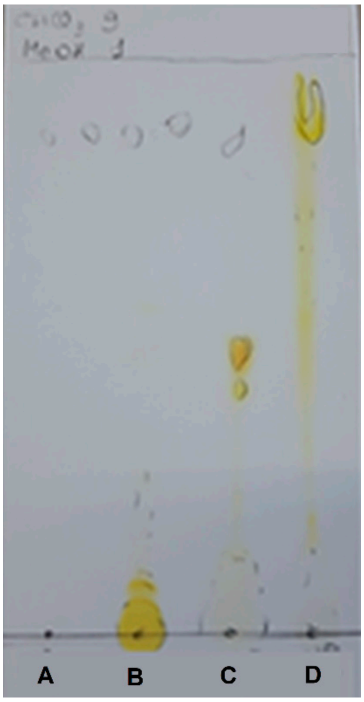

Figure S1. TLC plate with spots related to sericin (**A**), crocin (**B**), crocetin (**C**) and NPc (**D**).
